# Genome Sequence of the Euryhaline Javafish Medaka, *Oryzias javanicus*: A Small Aquarium Fish Model for Studies on Adaptation to Salinity

**DOI:** 10.1534/g3.119.400725

**Published:** 2020-01-27

**Authors:** Yusuke Takehana, Margot Zahm, Cédric Cabau, Christophe Klopp, Céline Roques, Olivier Bouchez, Cécile Donnadieu, Celia Barrachina, Laurent Journot, Mari Kawaguchi, Shigeki Yasumasu, Satoshi Ansai, Kiyoshi Naruse, Koji Inoue, Chuya Shinzato, Manfred Schartl, Yann Guiguen, Amaury Herpin

**Affiliations:** * Department of Animal Bio-Science, Faculty of Bio-Science, Nagahama Institute of Bioscience and Technology, 1266 Tamura, Nagahama 526-0829, Japan,; † Plate-forme bio-informatique Genotoul, Mathématiques et Informatique Appliquées de Toulouse, INRAE, Castanet Tolosan, France,; ‡ SIGENAE, GenPhySE, Université de Toulouse, INRAE, ENVT, Castanet Tolosan, France,; § INRAE, US 1426, GeT-PlaGe, Genotoul, Castanet-Tolosan, France,; ** MGX, Biocampus Montpellier, CNRS, INSERM, University of Montpellier, Montpellier, France,; †† Department of Materials and Life Sciences, Faculty of Science and Technology, Sophia University, 7-1 Kioi-cho Chiyoda-ku, Tokyo 102-8554, Japan,; ‡‡ Laboratory of Bioresources, National Institute for Basic Biology, 38 Nishigonaka, Myodaiji-cho, Okazaki 444-8585, Japan,; §§ Atmosphere and Ocean Research Institute, The University of Tokyo, 5-1-5 Kashiwanoha, Kashiwa 277-8564, Japan,; *** University of Wuerzburg, Developmental Biochemistry, Biocenter, 97074 Wuerzburg, Germany,; ††† Comprehensive Cancer Center Mainfranken, University Hospital, 97080 Wuerzburg, Germany,; ‡‡‡ Hagler Institute for Advanced Study and Department of Biology, Texas A&M University, College Station, Texas 77843, USA,; §§§ INRAE, UR 1037 Fish Physiology and Genomics, F-35000 Rennes, France, and; **** State Key Laboratory of Developmental Biology of Freshwater Fish, College of Life Sciences, Hunan Normal University, Changsha, 410081, Hunan, P. R. of China

**Keywords:** Medaka, evolution, whole genome sequencing, long reads, genetic map, transcriptome, adaptation, salinity

## Abstract

The genus *Oryzias* consists of 35 medaka-fish species each exhibiting various ecological, morphological and physiological peculiarities and adaptations. Beyond of being a comprehensive phylogenetic group for studying intra-genus evolution of several traits like sex determination, behavior, morphology or adaptation through comparative genomic approaches, all medaka species share many advantages of experimental model organisms including small size and short generation time, transparent embryos and genome editing tools for reverse and forward genetic studies. The Java medaka, *Oryzias javanicus*, is one of the two species of medaka perfectly adapted for living in brackish/sea-waters. Being an important component of the mangrove ecosystem, *O. javanicus* is also used as a valuable marine test-fish for ecotoxicology studies. Here, we sequenced and assembled the whole genome of *O. javanicus*, and anticipate this resource will be catalytic for a wide range of comparative genomic, phylogenetic and functional studies. Complementary sequencing approaches including long-read technology and data integration with a genetic map allowed the final assembly of 908 Mbp of the *O. javanicus* genome. Further analyses estimate that the *O. javanicus* genome contains 33% of repeat sequences and has a heterozygosity of 0.96%. The achieved draft assembly contains 525 scaffolds with a total length of 809.7 Mbp, a N50 of 6,3 Mbp and a L50 of 37 scaffolds. We identified 21454 predicted transcripts for a total transcriptome size of 57, 146, 583 bps. We provide here a high-quality chromosome scale draft genome assembly of the euryhaline Javafish medaka (321 scaffolds anchored on 24 chromosomes (representing 97.7% of the total bases)), and give emphasis on the evolutionary adaptation to salinity.

Medaka fishes belong to the genus *Oryzias* and are an emerging model system for studying the molecular basis of vertebrate evolution. This genus contains approximately 35 species, individually exhibiting numerous morphological, ecological and physiological differences and specificities (Inoue and Takei 2002, 2003; Parenti 2008; Mokodongan and Yamahira 2015). In addition, they all share many advantages of experimental model organisms, such as their small size, easy breeding, short generation time, transparent embryos, transgenic technology and genome-editing tools, with the “flag ship” species of this genus, the Japanese rice fish, *Oryzias latipes* (Wittbrodt *et al.* 2002; Kirchmaier *et al.* 2015). Such phenotypic variations, together with cutting edge molecular genetic tools make it possible to identify major loci that contribute to evolutionary differences, and to dissect the roles of individual genes and regulatory elements by functional tests. For example, a recent genetic mapping approach using interspecific hybrids identified the major chromosome regions that underlie the different hyperosmotic tolerance between species of the *Oryzias* genus (Myosho *et al.* 2018). Medaka fishes are also excellent models to study evolution of sex chromosomes and sex-determining loci among species (Takehana *et al.* 2007a, 2007b; Tanaka *et al.* 2007; Herpin and Schartl 2009), with the advantage of being also suitable models for providing functional evidences for these novel sex-determining genes by gain-of-function and/or loss-of-function experiments (Myosho *et al.* 2012; Takehana *et al.* 2014).

Among these species, the Java medaka, *Oryzias javanicus* (Figure 1), is unique as being the prototypic species of this genus with respect to adaptation to seawater. Previous phylogenetic studies divided the genus *Oryzias* into three monopyletic groups: *(i) javanicus*, *(ii) latipes* and *(iii) celebensis* species groups (Takehana *et al.* 2005; Mokodongan and Yamahira 2015). Most of the *Oryzias* species inhabit mainly freshwater biotopes, while only two species belonging to the *javanicus* group live in sea- or brackish water. One is *O. javanicus*, found in mangrove swamps from Thailand to Indonesia, and the other is *O. dancena* (previously named *O. melastigma*) living both in sea- and freshwaters from India to Malaysia. Although both species are highly adaptable to seawater, *O. javanicus* prefers hyperosmotic conditions while *O. dancena* favors hypoosmotic conditions at the west coast of Malaysian peninsula where their distribution ranges overlap (Yusof *et al.* 2012). In addition, *O. javanicus* is an important component of the mangrove ecosystem (Zulkifli *et al.* 2012), and has been used as a valuable marine test fish in several ecotoxicology studies (Koyama *et al.* 2008; Horie *et al.* 2018).

**Figure 1:**
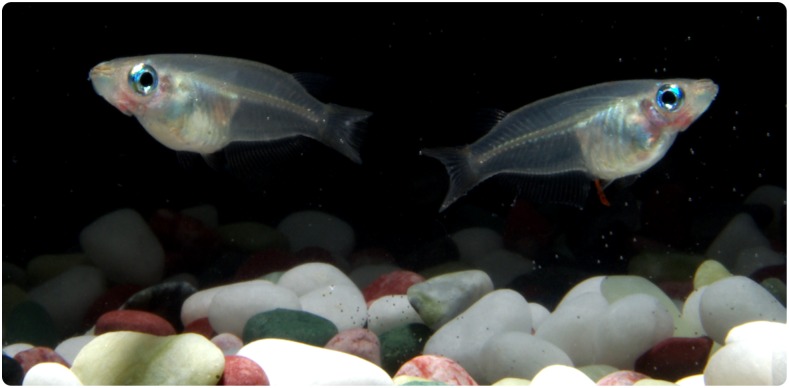
A couple of Java medakas, Oryzias javanicus. Picture from K. Naruse, NBRP Medaka stock center (https://shigen.nig.ac.jp/medaka/top/top.jsp).

In this study, we sequenced and assembled the whole genome of *O. javanicus*, a model fish species for studying molecular mechanisms of seawater adaptation. In teleost fish, the major osmoregulatory organs *i.e.*, gills, intestine and kidney, play different roles for maintaining body fluid homeostasis. Many genes encoding hormones, receptors, osmolytes, transporters, channels and cellular junction proteins are potentially involved in this osmotic regulation. In addition to osmoregulation, hatching enzyme activity dramatically fluctuates and adjusts at different salt conditions. At hatching stage, fish embryos secrete a specific cocktail of enzymes in order to dissolve the egg envelope, or chorion. In the medaka *O. latipes*, digestion of the chorion occurs through the cooperative action of two kinds of hatching enzymes, ***(i)*** the high choriolytic enzyme (HCE) and ***(ii)*** the low choriolytic enzyme (LCE) (Yasumasu *et al.* 2010). The HCE displays a higher activity in fresh- than in brackish waters (Kawaguchi *et al.* 2013). Thus, availability of a high-quality reference genome in *O. javanicus* would facilitate further research for investigating the molecular basis of physiological differences, including the osmotic regulation and the hatching enzyme activity, among *Oryzias* species.

## Methods and Materials

### Animal samplings

The wild stock of *O. javanicus* used in this study was supplied by the National Bio-Resource Project (NBRP) medaka in Japan. This stock (strain ID: RS831) was originally collected at Penang, Malaysia, and maintained in synthetic seawater (ca 3% of NaCl equivalent; although using half seawater is also possible) in aquaria under an artificial photoperiod of 14 hr light:10 hr darkness at 27 ± 2°. Genomic DNA was extracted from the whole body of a female (having ZW sex chromosome) using a conventional phenol/chloroform method, and was subjected to PacBio and 10X Genomics sequencings. For RNA-sequencing, total RNAs were extracted from nine female tissues (brain, bone, gill, heart, intestine, kidney, liver, muscle and ovary), and one male tissue (testis) using the RNeasy Mini Kit (Qiagen). For genetic mapping, we used a DNA panel consisting of 96 F1 progeny with their parents (originally described in a previous study (Takehana *et al.* 2008)). Phenotypic sex was determined by secondary sex characteristics of adult fish (six-month-old and sexually mature fish), namely, the shapes of dorsal and anal fins. All animal experiments performed in this study complied with the guideline of National Institute for Basic Biology, and have been approved by the Institutional Animal Care and Use Committee of National Institute of Natural Science (16A050 and 17A048).

#### PacBio genome sequencing:

Library construction and sequencing were performed according to the manufacturer’s instructions (Shared protocol-20kb Template Preparation Using BluePippin Size Selection system (15kb size Cutoff)). When required, DNA was quantified using the Qubit dsDNA HS Assay Kit (Life Technologies). DNA purity was assessed by spectrophotometry using the nanodrop instrument (Thermofisher), and size distribution and absence of degradation were monitored using the Fragment analyzer (AATI) (8–11). Purification steps were performed using 0.45X AMPure PB beads (PacBio). 80µg of DNA was purified and then sheared at 40kb using the megaruptor system (diagenode). DNA and END damage repair step was further performed for 5 libraries using the SMRTBell template Prep Kit 1.0 (PacBio). Blunt hairpin adapters were then ligated to the libraries. Libraries were subsequently treated with an exonuclease cocktail in order to digest unligated DNA fragments. Finally, a size selection step using a 15kb cutoff was performed on the BluePippin Size Selection system (Sage Science) using 0.75% agarose cassettes, Marker S1 high Pass 15-20kb. Conditioned sequencing primer V2 was annealed to the size-selected SMRTbell. The annealed libraries were then bound to the P6-C4 polymerase using a ratio of polymerase to SMRTbell set at 10:1. After performing a magnetic bead-loading step (OCPW), SMRTbell libraries were sequenced on 48 SMRTcells (RSII instrument at 0.25nM with a 360-min movie resulting in a total of 61.8Gb of sequence data (1.28Gb/SMRTcell).

#### 10X Genomics genome sequencing:

Chromium library was prepared according to 10X Genomics’ protocol using the Genome Reagent Kits v1. Sample quantity and quality controls were further validated on Qubit, Nanodrop and Femto. Optimal performance has been characterized on input gDNA with a mean length greater than 50 kb. The library was prepared using 3 µg of high molecular weight (HMW) gDNA (cut off at 50kb using BluePippin system). In details, for the microfluidic Genome Chip, a library of Genome Gel Beads was combined with HMW template gDNA in Master Mix and partitioning oil in order to create Gel Bead-In-EMulsions (GEMs) in the Chromium. Each Gel Bead was functionalized with millions of copies of a 10x Barcoded primer. Upon dissolution of the Genome Gel Bead in the GEM, primers containing (*i*) an Illumina R1 sequence (Read 1 sequencing primer), (*ii*) a 16 bp 10x Barcode, and (*iii*) a 6 bp random primer sequence were released. Read 1 sequence and the 10x Barcode were added to the molecules during the GEM incubation. P5 and P7 primers, Read 2, and Sample Index were added during library construction. 8 cycles of PCR were performed for amplifying the library. Library quality was assessed using a Fragment analyzer. Finally, the library was sequenced on an Illumina HiSeq3000 using a paired-end read length of 2x150 pb with the Illumina HiSeq3000 sequencing kits resulting in 101.6Gb of raw sequence data.

#### Genome assembly and annotation:

**Figure 2:**
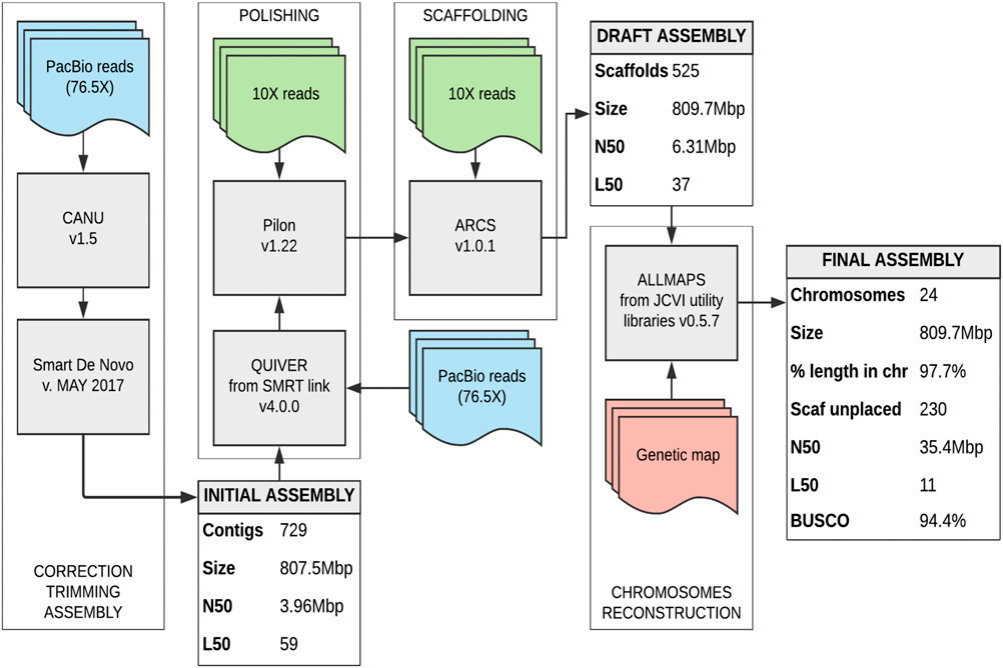
Oryzias javanicus assembly pipeline. Sequencing data are represented by colored rectangles with waved bases. Tools used are in gray rectangles. Assembly metrics are in gray and white rectangles. This pipeline is divided in stages symbolized by the frame.

PacBio reads were corrected and trimmed using Canu v1.5 (Koren *et al.* 2017). Contigs were then assembled using SMARTdenovo version of May 2017 (Ruan 2019). The draft assembly produced contains 729 contigs with a total genome size of 807.5 Mbp, an N50 of 3,9 Mbp and a L50 of 59 contigs (Figure 2). To improve the assembly base pair quality two polishing steps were run. First, BLASR aligned PacBio reads were processed with Quiver from the Pacific Biosciences SMRT link software v.4.0.0. Second, 10X reads were realigned to the genome using Long Ranger v2.1.1 and the alignment file was processed with Pilon v1.22 (Walker *et al.* 2014). Third, the same 10X reads were aligned to the genome with BWA-MEM v0.7.12-r1039 (Li 2013) and the alignment file was processed with ARCS v1.0.1 (Yeo *et al.* 2018) to scaffold the genome. Both tools were run with default parameters. For genome annotation, the MAKER3 pipeline was employed ((Holt and Yandell 2011); Maker 3.01.02-beta in mpi mode to merge data from gene models and cDNA/protein evidences). Maker has been running with entries est_gff, protein_gff and pred_gff in run_evm = 1, est2genome = 0 and protein2genome = 0 mode. No AED cut-off was applied but AED scores have been used to select the best supported transcript for each gene.

#### Transcriptome RNA-seq sequencing and assembly:

RNA-seq libraries were prepared according to Illumina’s protocols using the Illumina TruSeq Stranded mRNA sample prep kit. Briefly, mRNAs were selected using poly-T beads, reverse-transcribed and fragmented. The resulting cDNAs were then subjected to adaptor ligation. 10 cycles of PCR were performed for amplifying the libraries. Quality of the libraries was assessed using a Fragment Analyzer. Quantification was performed by qPCR using the Kapa Library Quantification Kit. RNA-seq libraries were sequenced on an Illumina HiSeq3000 using a paired-end read length of 2x150 pb with the Illumina HiSeq3000 sequencing kits resulting in 95Gb of sequence data (28.9M reads pairs/library). The read quality of the RNA-seq libraries was evaluated using FastQC (Andrew S. 2010). *De novo* and reference-based transcriptome assemblies were produced. Reads were cleaned, filtered and *de novo* assembled using the DRAP pipeline v1.91 (Cabau *et al.* 2017) with the Oases assembler (Schulz *et al.* 2012). Assembled contigs were filtered in order to keep only those with at least one fragment per kilobase of transcript per million reads (FPKM). In the reference-based approach, all clean reads were mapped to the chromosomal assembly using STAR v2.5.1b (Dobin *et al.* 2013) with outWigType and outWigStrand options to output signal wiggle files. Cufflinks v2.2.1 (Trapnell *et al.* 2010) was used to assemble the transcriptome. All tissues have been *de novo* assembled separately and the 1 FPKM cut-off has been set on each library. The min, mean, median and max number of transcripts after the cufflinks assembly of each tissue are respectively 26223, 44992, 47804 and 59234.

#### RAD-library construction:

RAD-seq library was built following the Baird *et al.* (Baird *et al.* 2008) protocol with minor modifications. Briefly, between 400 to 500 ng of gDNA per fish were digested with SbfI-HF enzyme (R3642S, NEB). Digested DNA was purified using AMPure PX magnetic beads (Beckman Coulters) and ligated to indexed P1 adapters (1 index per sample) using concentrated T4 DNA ligase (M0202T, NEB). After quantification (Qubit dsDNA HS assay kit, Thermofisher) all samples were pooled in equal amounts. The pool was then fragmented on a S220 sonicator (Covaris) and purified with Minelute column (Qiagen). Finally, the sonicated DNA was size selected (250 to 450 bps) on a Pippin HT (Sage science) using a 2% agarose cassette, repaired using the End-It DNA-end repair kit (Tebu Bio) and adenylated at its 3′ ends using Klenow (exo-) (Tebu-Bio). P2 adapters were then ligated using concentrated T4 DNA ligase, and 50 ng of the ligation product were engaged in a 12 cycles PCR for amplification. After AMPure XP beads purification, the resulting library was checked on a Fragment Analyzer (Agilent) using the HS NGS kit (DNF-474-33) and quantified by qPCR using the KAPA Library Quantification Kit (Roche, ref. KK4824). Ultimately the whole library was denatured, diluted to 10 pM, clustered and sequenced using the rapid mode v2 SR100nt lane of a Hiseq2500 device (Illumina).

### Identification of genes associated with osmoregulation

We listed known *O. latipes* genes that encode proteins associated with osmoregulation (*e.g.*, hormones, pumps, transporters, channels, osmolytes-related and cellular junction proteins) based on literatures, and used HMMER version 3.1b2 (http://hmmer.org/) to identify specific Pfam domains (Pfam 32, (El-Gebali *et al.* 2019) included in these proteins (Supplemental Table S1). Using our gene model of *O. javanicus* together with Ensembl gene models of the *O. dancena* and *O. latipes* species complex (Hd-rR, HNI-II and HSOK), we counted the number of proteins containing the Pfam domains in each species (Supplemental Table S2).

#### Salt dependency of OjHCE:

The mature enzyme regions of OjHCE3 was amplified from their full-length cDNA using primers designed to contain suitable restriction enzyme sites (BamHI and NdeI) at the 5′ region. After digestion with BamHI and NdeI, the fragments were inserted into pET3c vector. The plasmid was transformed into *E. coli* BL21 (DE3) pLysE strain cells. The cells were cultivated, and recombinant protein was harvested as inclusion body as described in (Kawaguchi *et al.* 2013). After the inclusion body was dissolved in denaturing buffer (50 mM Tris-HCl (pH 8.0), 8 M urea, 0.1 M 2-mercaptoethanol and 1 mM EDTA), the recombinant protein was refolded as described in (Kawaguchi *et al.* 2013). The egg envelope digestion activity of OjHCE was determined by turbidimetric methods (Yamagami 1973; Kawaguchi *et al.* 2013). The isolated egg envelopes of medaka *O. latipes* were minced into fine fragments, and suspended in distilled water (DW). The suspension was allowed to stand overnight to remove rough fragments, and the supernatant containing fine fragments was used as substrate. The enzyme reaction was carried out in 400 μl of a reaction mixture containing 50 mmol/l Tris-HCl (pH 8.0), 0−0.75 mol/l NaCl, egg envelope suspension and recombinant HCE. The initial turbidity at 610 nm (T610) of the mixture was adjusted to approximately 55% when that of DW was 100%. Increment in transmission caused by the digestion of the fragmented envelopes was monitored for 3 min. The relative enzyme activity was expressed as the percentage of the highest activity under various salt concentrations.

### Data availability

All genome and transcriptome information was deposited under the NCBI Bioproject number PRJNA505405. The Illumina sequencing data for the RAD-tag genetic map were deposited in the Sequence Read Archive at NCBI with accession numbers SRX5326271 to SRX5326366. The genomic PacBio sequencing data were deposited in the Sequence Read Archive at NCBI with accession numbers SRX5274121 to SRX5274138 and SRX5274139 to SRX5274169. The 10X genomics Illumina sequencing data were deposited in the Sequence Read Archive at NCBI with accession number SRX5274139. The transcriptome Illumina sequencing data were deposited in the Sequence Read Archive at NCBI with accession numbers SRX5017469 to SRX5017479. The final chromosome assembly and genome annotation were deposited in GenBank at NCBI RWID00000000.1. Supplemental material available at figshare: https://doi.org/10.25387/g3.10310498.

## Results and Discussion

### Genome Characteristics

To estimate size and other genome characteristics, 10X reads were processed with Jellyfish v1.1.11 (Marçais and Kingsford 2011) to produce 21-mer distribution. The k-mer histogram was uploaded to GenomeScope (Vurture *et al.* 2017) with the max k-mer coverage parameter set to 10,000. Genome size was estimated around 908 Mbp, which is slightly higher than the 850 Mbp (0.87pg) estimated size reported on the Animal Genome Size Database (“Animal Genome Size Database:: Home”). Furthermore, this analysis estimates that the *O. javanicus* genome contains 33% of repeat sequences (around 303 Mbp) and has a heterozygosity of 0.96% (Table 1).

**Table 1:** GenomeScope outputs on *O. javanicus* genome statistics

Property	min	max
**Heterozygosity**	0.960%	0.964%
**Genome Haploid Length**	908,146,324 bp	908,641,143 bp
**Genome Repeat Length**	303,610,795 bp	303,776,222 bp
**Genome Unique Length**	604,535,529 bp	604,864,921 bp
**Model Fit**	95.95%	99.72%
**Read Error Rate**	1.50%	1.50%

### Genome assembly

Draft assembly contains 525 scaffolds with a total length of 809.7 Mbp, a N50 of 6,3 Mbp and a L50 of 37 scaffolds. This represents 89.1% of the k-mer estimated genome size. Given the high percentage of repeats in the *O. javanicus* genome (33%), it is possible that the PacBio assembly did not totally succeed in completing all repeated regions. The genome completeness was estimated using Benchmarking Universal Single-Copy Orthologs (BUSCO) v3.0 (Simão *et al.* 2015) based on 4,584 BUSCO orthologs derived from the Actinopterygii lineage leading to BUSCO scores of 4,327 (94.4%) complete BUSCOs, 176 (3.8%) fragmented BUSCOs and 81 (1.8%) missing BUSCOs.

### Integration with the genetic map

RAD reads were trimmed by Trim Galore 0.4.3 (“Trim Galore”) with Cutadapt 1.12 (Martin 2011) and then mapped to the assembled scaffolds using BWA-MEM v0.7.17 (Li 2013). Uniquely mapped reads were extracted from the read alignments, and then called variant bases using uniquely mapped reads by samtools *mpileup* and bcftools *call* (Li 2011). Indels and variants with a low genotyping quality (GQ < 20), a low read depth (DP < 5), a low frequency of the minor allele (< 5%), more than four alleles in the family, no more than 5% individuals missing were removed by vcftools v0.1.15 (Danecek *et al.* 2011). After quality filtering, 6,375 variant sites were kept for the following analysis. Linkage map was constructed using this genotype information using Lep-MAP3 (Rastas 2017). Briefly, the filtered vcf file was loaded and the markers removed with high segregation distortion (*Filtering2*: dataTolerance = 0.001). Markers were then separated into 24 linkage groups with a LOD score threshold set at 9 and a fixed recombination fraction of 0.08 (*SeparateChromosomes2*: lodlimit = 9 and theta = 0.08). Two linkage groups were then excluded because of their small numbers of contained markers (less than 10). Classification of the markers was determined after maximum likelihood score indexing with 100 iterations (*OrderMarkers2*: numMergeIterations = 100) in each linkage group. The final map had 5,738 markers dispatched among 24 linkage groups spanning a total genetic distance of 1,221 cM.

The linkage map exhibited discrepancies between genomic scaffolds and genetic markers. Among 525 genomics scaffolds, 32 were linked to more than one linkage group. To split chimeric scaffolds with a higher precision and to rebuild chromosomes with a higher fidelity, we used a cross-species synteny map between the Java medaka (*O. javanicus*) scaffolds and the medaka *(O. latipes*) chromosomes in order to combine marker locations from genetic and synteny maps. To build the synteny map, medaka cDNAs were aligned to the Java medaka scaffolds using BLAT v36 (Kent 2002), and a list of pairwise correspondence of gene positions on Java medaka scaffolds and medaka chromosomes was established. 13,796 markers were added to the 5,738 markers of the genetic map. Java medaka chromosomes were then reconstructed using ALLMAPS from the JCVI utility libraries v0.5.7 (Tang *et al.* 2015). This package was used to combine genetic and synteny maps, to split chimeric scaffolds, to anchor, order and orient genomic scaffolds. The resulting chromosomal assembly consists of 321 scaffolds anchored on 24 chromosomes (97.7% of the total bases) and 231 unplaced scaffolds

### Annotation results

The first annotation step was identifying repetitive DNA content using RepeatMasker v4.0.7 (“RepeatMasker Home Page”), Dust (Morgulis *et al.* 2006) and TRF v4.09 (Benson 1999). A species-specific *de novo* repeat library was built with RepeatModeler v1.0.11 (Smit and Hubley 2010). Repeated regions were located using RepeatMasker with the *de novo* and the Zebrafish (*Danio rerio*) libraries. Bedtools v2.26.0 (Quinlan and Hall 2010) was used to merge repeated regions identified with the three tools and to soft mask the genome. Repeats were estimated to account for 43.16% (349 Mbp) of our chromosomal assembly. The MAKER3 genome annotation pipeline v3.01.02-beta (Holt and Yandell 2011) combined annotations and evidences from three approaches: similarity with known fish proteins, assembled transcripts and *de novo* gene predictions. Protein sequences from 11 other fish species (*Astyanax mexicanus*, *Danio rerio*, *Gadus morhua*, *Gasterosteus aculeatus*, *Lepisosteus oculatus*, *Oreochromis niloticus*, *Oryzias latipes*, *Poecilia formosa*, *Takifugu rubripes*, *Tetraodon nigroviridis*, *Xiphophorus maculatus*) found in Ensembl were aligned to the masked genome using Exonerate v2.4 (Slater and Birney 2005). Previously assembled transcripts were used as RNA-seq evidence. A *de novo* gene model was built using Braker v2.0.4 (Hoff *et al.* 2016) with wiggle files provided by STAR as hints file for training GeneMark and Augustus. The best supported transcript for each gene was chosen using the quality metric Annotation Edit Distance (AED) (Eilbeck *et al.* 2009). The genome annotation gene completeness was assessed by BUSCO using the Actinopterygii group (Table 2). Finally, the predicted genes were subjected to similarity searches against the NCBI NR database using Diamond v0.9.22 (Buchfink *et al.* 2015). The top hit with a coverage over 70% and identity over 80% was retained.

**Table 2:** Java medaka assembly and annotation statistics

Gene annotation	
Number of genes	21,454
Number of transcripts	21,454
Transcriptome size	57,146,583 bp
Mean transcript length	2,663 bp
Longest transcript	42,733 bp
Number of genes with significant hit against NCBI NR	17,412 (81.2%)
**Gene completeness**	
Complete BUSCOs	4,289 (93.6%)
Fragmented BUSCOs	187 (4.1%)
Missing BUSCOs	108 (2.3%)

### Mitochondrial genome and annotation

The previously sequenced *Oryzias javanicus* mitochondrial genome (NC_012981) (Setiamarga *et al.* 2009) was aligned to the chromosomal assembly using Blat. All hits were supported by a single scaffold. This scaffold was removed from the assembly, circularised and annotated using MITOS (Bernt *et al.* 2013). This new *Oryzias javanicus* mitochondrial genome is 16,789 bp long and encodes 13 genes, 2 rRNAs and 19 tRNAs.

#### Phylogenetic relationship:

**Figure 3:**
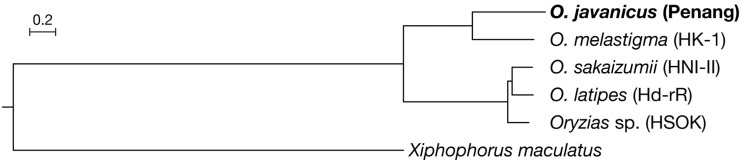
Phylogenetic position of *O*. *javanicus*. Maximum likelihood tree was inferred from the concatenated codon-alignment of 10,852 single-copy genes among 5 reference assemblies of *Oryzias* species with Southern platyfish (*Xiphophorus maculatus*) as outgroup. All nodes were supported by 100% bootstrap values.

To precisely determine the phylogenetic position of *O. javanicus* within the genus *Oryzias*, we estimated the phylogenetic relationship using published whole genome datasets as references. Reference assemblies and annotations of *O. latipes* (Hd-rR: ASM223467v1), *O. sakaizumii* (HNI-II: ASM223471v1), *Oryzias* sp. (HSOK: ASM223469v1), *O. dancena* (Om_v0.7.RACA), and southern platyfish *Xiphophorus maculatus* (X_maculatus-5.0-male) were obtained from Ensembl Release 94 (http://www.ensembl.org/). Among the six genomes, orthologous groups were classified and 10,852 single-copy orthologous genes were identified using OrthoFinder 2.2.6 (Emms and Kelly 2015). For every single gene, codon alignment based on translated peptide sequences was generated by PAL2NAL (Suyama *et al.* 2006) and then trimmed by trimAl with ‘-autometed1’ option (Capella-Gutiérrez *et al.* 2009). All multi-sample fasta files were concatenated into a single file using AMAS *concat* by setting each gene as a separate partition (Borowiec 2016). A maximum likelihood tree was then inferred using IQ-TREE v1.6.6 (Nguyen *et al.* 2015) with the GTR+G substitution model for each codon, followed by an ultrafast bootstrap analysis of 1,000 replicates (Hoang *et al.* 2018). This tree (Figure 3) indicates that *O. javanicus* forms a monophyletic group with *O. dancena* but not with the *O. latipes* species complex (Hd-rR, HNI-II, and HSOK), being consistent with previous trees inferred from two mitochondrial genes and a nuclear gene (Takehana *et al.* 2005).

**Figure 4:**
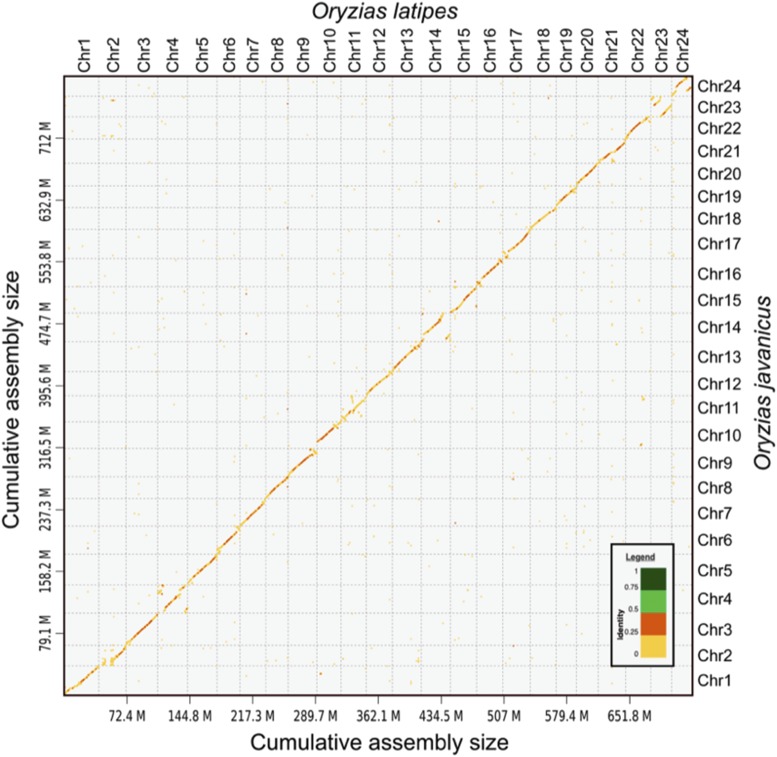
Genome-wide comparison of the *Oryzias javanicus* and *Oryzias latipes* genome assemblies. This dot-plot alignment of the *O. javanicus* genome with the *O. latipes* reference genome [Ensembl version ASM223467v1 (GCA_002234675.1)] was produced with the D-GENIES software (Cabanettes and Klopp 2018) available online at http://dgenies.toulouse.inra.fr/ with default visualization parameters and a slight filtering of the small matches. The different ranges of percentage of identity are shown in different colors (legend panel with the identity percentage color range).

The D-GENIES (Cabanettes and Klopp 2018) genome-wide comparison of this *O. javanicus* genome compared to the *O. latipes* reference genome [Ensembl version ASM223467v1 (GCA_002234675.1)] shows that these two genomes are extremely colinear at the whole genome scale (Figure 4). At a chromosome scale the comparison with *O. latipes* shows that most of the *O. javanicus* chromosomes are strongly colinear with their single *O. latipes* chromosome counterparts (Supplemental Figure 1). Only a few *O. Javanicus* chromosomes are more deeply reorganized compared to their *O. latipes* chromosome counterparts with for instance, the *O. javanicus* LG04, LG14, LG23 and LG24 that display multiple intra chromosomal rearrangements and the *O. javanicus* LG10, LG11 and LG14 that show small inter chromosomal rearrangements (*i.e.*, the insertion of a small region from an *O. latipes* different chromosome). With regards to their sex chromosomes *O. latipes* has a male heterogametic system (XX/XY) and the LG01 is the Y sex chromosome (see (Herpin and Schartl 2009) for review). *O. javanicus* has a female heterogametic sex determination system and LG16 is the W sex chromosome (Takehana *et al.* 2008). In *O. javanicus* both the LG01 (the *O. latipes* Y chromosome) and the LG16 (the *O. javanicus* W chromosome) display a strong chromosome collinearity with respectively the *O. latipes* LG01 and LG16. Indeed, and according to previous reports (Takehana *et al.* 2008) the *dmrt1bY* Y specific duplication/insertion on *O. latipes* LG01 is absent from the *O. javanicus* LG01 sequence.

### Adaptation to salinity and hatching enzymes

To gain insight into gene family evolution associated with osmoregulation, we used HMMER version 3.1b2 to identify Pfam domain containing proteins in the *O. javanicus* genome. We used protein sequences based on our gene model of *O. javanicus* combined with Ensembl genes of the *O. latipes* species complex (Hd-rR, HNI-II and HSOK) and *O. dancena* for the Pfam search, and focused on 147 domains found in 224 proteins whose functions were related to osmoregulation (Supplemental Tables S1 and S2). Similar numbers of proteins were observed among species for each domain, suggesting that the osmoregulation gene repertoires are relatively conserved in *Oryzias* species. However, further detailed comparisons are required because gene annotation methods are different among data.

**Figure 5:**
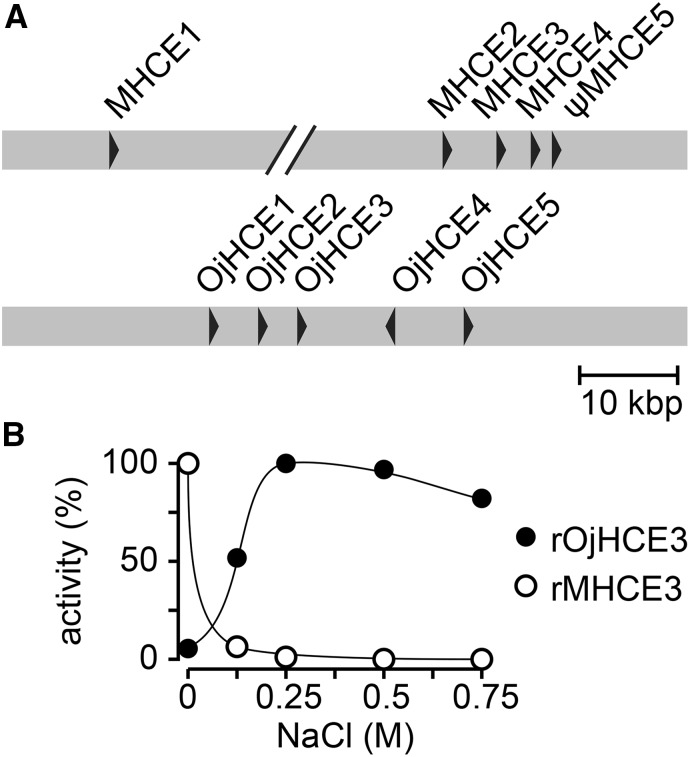
Hatching enzyme of Oryzias javanicus. (A) *hce* gene cluster of *O. latipes* (*mhce1-5*) and *O. javanicus* (*Ojhce1-5*). Arrowheads indicate direction of transcription. (B) Salt dependency of *O. javanicus hce* (black circle) and *O. latipes* (white circle). Activities are shown as % of relative activity with respect to highest activity, which is considered as 100% in each species.

We then also focused on specific genes encoding hatching enzymes. In the genome of *O. latipes*, five copies of *hce* genes -including one pseudogene- are clustered tandemly with the same transcriptional direction on chromosome 3 (chr. 3), while only one single copy of the *lce* gene is located on chromosome 24 (chr. 24) (Kawaguchi *et al.* 2007). In *O. javanicus* 5 copies of the *hce (Ojhce)* gene are located on chromosome 3 and one *lce (Ojlce)* gene was found on chromosome 24. The amino acid sequence similarities in the mature enzyme region of the 5 *Ojhce* genes are between 89–99%. Only in comparison to *O. latipes*, within the five *O. javanicus hce* genes, the fourth one (*Ojhce4*) displays an opposite orientation compared to the others (Figure 5A) suggesting a re-arrangement within the *hce* gene cluster that has likely been occurring during the evolution of *Oryzias* lineage. Phylogenetic analyses indicated that all the cloned *hce* and *lce* genes were orthologous to other euteleosteans *hce* and *lce* respectively.

While LCE’s activity remains constant over various salinities, HCEs have been reported to show salt-dependent activity (Kawaguchi *et al.* 2013). In contrast to other *Oryzias* species, *O. javanicus*, being a euryhaline species, specifically adapted its physiology to higher water salinities. In order to test whether such adaptive evolution would translate at the level of HCE activity, recombinant OjHCE3 (rOjHCE3) was generated in an *E. coli* expression system, refolded, and its activity regarding to the digestion of the egg-envelope determined at various salt concentrations based on the method described in Kawaguchi *et al.* (Kawaguchi *et al.* 2013). Although rOjHCE3 showed virtually no activity at 0 M NaCl, an increased activity was apparent at elevated salt concentrations. Furtheron rOjHCE3 activity was recorded to be highest at 0.25 M NaCl, while still maintaining high activity up to 0.75 M NaCl (Figure 5B). In contrast, it has been reported that *O. latipes* HCEs show highest activity at 0 M NaCl, and drastically decrease when salt concentrations increase ((Kawaguchi *et al.* 2013), Figure 5B). These results suggest that salt preference of HCE enzymes is a species-specific adaptation to different salt environments at hatching.

The Java medaka, *Oryzias javanicus*, is one of the two species of medaka living in brackish/sea-waters. Being an important component of the mangrove ecosystem, *O. javanicus* is also used as a valuable marine test-fish for ecotoxicology studies. Here, we sequenced and assembled the whole genome of *O. javanicus*. Complementary sequencing approaches and data integration with a genetic map allowed the final assembly of the 908 Mbp of the *O. javanicus* genome. The final draft assembly contains 525 scaffolds with a total length of 809.7 Mbp, a N50 of 6,3 Mbp and a L50 of 37 scaffolds. Providing here a high-quality draft genome assembly of the euryhaline Javafish medaka, we anticipate this resource will be catalytic for a wide range of comparative genomic, phylogenetic and functional studies within the genus *Oryzias* and beyond.
